# Enhanced sensitivity of laforin- and malin-deficient mice to the convulsant agent pentylenetetrazole

**DOI:** 10.3389/fnins.2014.00291

**Published:** 2014-09-12

**Authors:** Ana M. García-Cabrero, Gentzane Sánchez-Elexpuru, José M. Serratosa, Marina P. Sánchez

**Affiliations:** ^1^Laboratory of Neurology, IIS-Fundación Jiménez Díaz, Universidad Autónoma de MadridMadrid, Spain; ^2^Centro de Investigación Biomédica en Red de Enfermedades RarasMadrid, Spain

**Keywords:** Lafora disease, epilepsy, *Epm2a^−/−^* and *Epm2b^−/−^* mice, PTZ, seizure threshold

## Abstract

Lafora disease is a rare form of inherited progressive myoclonus epilepsy caused by mutations in the *EPM2A* gene encoding laforin, or in the *EPM2B* gene, which encodes malin. It is characterized by the presence of polyglucosan inclusion bodies (Lafora bodies) in brain and other tissues. Genetically engineered mice lacking expression of either the laforin (*Epm2a^−/−^*) or malin (*Epm2b^−/−^*) genes display a number of neurological and behavioral abnormalities that resemble those found in patients suffering from Lafora disease; of these, both *Epm2a^−/−^* and *Epm2b^−/−^* mice have shown altered motor activity, impaired motor coordination, episodic memory deficits, and different degrees of spontaneous epileptic activity. In this study, we analyze the sensitivity of *Epm2a^−/−^* and *Epm2b^−/−^* mice to the convulsant drug pentylenetetrazol (PTZ), an antagonist of the γ-aminobutyric acid type A (GABA_A_) receptor, commonly used to induce epileptic tonic-clonic seizures in laboratory animals. PTZ-induced epileptic activity, including myoclonic jerks and tonic-clonic seizures, was analyzed in 2 age groups of mice comprising representative samples of young adult and aged mice, after administration of PTZ at sub-convulsive and convulsive doses. *Epm2a^−/−^* and *Epm2b^−/−^* mice showed a lower convulsive threshold after PTZ injections at sub-convulsive doses. A lower convulsive threshold and shorter latencies to develop epileptic seizures were observed after PTZ injections at convulsive doses. Different patterns of generalized seizures and of discharges were observed in *Epm2a^−/−^* and *Epm2b^−/−^* mice. *Epm2a^−/−^* and *Epm2b^−/−^* mice present an increased sensitivity to the convulsant agent PTZ that may reflect different degrees of increased GABA_A_ receptor-mediated hyperexcitability.

## Introduction

Lafora disease (LD) is a rare form of inherited progressive myoclonus epilepsy (OMIM#254780; ORPHA501). Recessive mutations in either the *EPM2A* gene encoding a dual-specificity phosphatase known as laforin (OMIM 607566) (Minassian et al., 1998; Serratosa et al., 1999) or in the *EPM2B/NHLRC1* gene encoding malin (OMIM 608072), an E3 ubiquitin ligase (Chan et al., 2003; Gentry et al., 2005; Gomez-Abad et al., 2005; Singh et al., 2005, 2006) are responsible for the disease, although the existence of a third, minor locus has also been postulated (Chan et al., 2004).

Patients with LD develop progressive myoclonus epilepsy that usually starts in adolescence and includes absence, visual, myoclonic, and tonic-clonic seizures accompanied by rapid neurologic degeneration, including ataxia, dementia, dysarthria, amaurosis, respiratory failure, and a final vegetative state and death, usually within 10 years of onset (Lafora, 1911; Van Heycop Ten Ham, 1974; Berkovic et al., 1986, 1991; Roger et al., 1992; Acharya et al., 1995). The principal pathologic feature of LD is the presence of periodic acid-Schiff (PAS)-positive intracellular inclusions of polyglucosans (Lafora bodies) located mainly in the brain, the skeletal muscle, the heart, and the liver (Lafora, 1911; Lafora and Glueck, 1911; Harriman et al., 1955; Yokoi et al., 1968; Sakai et al., 1970; Carpenter and Karpati, 1981; Berkovic et al., 1986). Currently, no preventive or curative therapies exist for LD.

Different mouse models have been generated by disrupting either the *Epm2a* (Ganesh et al., 2002) or the *Epm2b* gene (Depaoli-Roach et al., 2010; Turnbull et al., 2010; Valles-Ortega et al., 2011; Criado et al., 2012). Of these, the *Epm2a^−/−^* mouse line generated by Ganesh et al. (2002) displays many of the neurological and behavioral abnormalities found in LD patients, including neuronal degeneration and the development of Lafora bodies in different organs including heart, liver, muscle, and a variety of brain regions such as hippocampus, cerebral cortex, thalamus, cerebellum, and brainstem (Ganesh et al., 2002). In addition, the *Epm2b^−/−^* mouse line previously generated by our group (Criado et al., 2012) also shows substantial neurological abnormalities that correlated with the detection of Lafora bodies in the same brain regions as those of the *Epm2a^−/−^* mouse line (Criado et al., 2012). Of those neurological abnormalities, both laforin-deficient and malin-deficient mouse lines showed altered motor activity, impaired motor coordination, episodic memory deficits, and different degrees of spontaneous epileptic activity, such as spontaneous single spikes, polyspikes and spike-wave and polyspike-wave complexes correlating with myoclonic jerks (Ganesh et al., 2002; Criado et al., 2012; Garcia-Cabrero et al., 2012). *Epm2a^−/−^* and *Epm2b^−/−^* mice had spontaneous tonic-clonic seizures, although the *Epm2b^−/−^* mice did not display EEG correlates (Garcia-Cabrero et al., 2012).

Here we analyze the responses of both *Epm2a^−/−^* and *Epm2b^−/−^* mice to the administration of the epileptogenic agent pentylenetetrazole (PTZ). PTZ is widely used for the study of epileptiform activity in laboratory animals (Vernadakis and Woodbury, 1969; Reinhard and Reinhard, 1977; Swinyard et al., 1989), and is also used routinely to test antiepileptic drugs (Swinyard et al., 1989). PTZ is an antagonist of the type A receptor of γ-aminobutyric acid (GABA_A_) (Stone, 1970). As with other GABA_A_ antagonists, administration of low doses of PTZ can result in absence seizures in animal models (Snead et al., 2000), whereas higher doses result in generalized tonic-clonic seizures (Woodbury et al., 1980; Panagopoulos et al., 1998; Coimbra et al., 2001a,b; Erakovic et al., 2001; Eloqayli et al., 2003). In this study, we used PTZ at both sub-convulsive and convulsive doses to analyze seizure thresholds in an attempt to elucidate the molecular mechanisms underlying altered neuronal excitability in *Epm2a^−/−^* and *Epm2b^−/−^* mice and to further evaluate novel therapeutic strategies to treat epileptic symptoms. PTZ induces seizure development shortly after injection (Pitkanen et al., 2006), with reversible effects and low toxicity at convulsive doses (Velisek, 2006), whereas other seizure inducing agents such as methionine sulfoximine or kainate (Dudek et al., 2006; Eid et al., 2008; Cloix and Hevor, 2009), induce development of seizures with long pre-convulsive periods of several hours, and others produce therapy-resistant seizures, e.g., strichnine (Löscher, 1997). This makes PTZ the most appropriate agent for seizure monitoring in our models.

## Materials and methods

### Experimental animals

Generation of *Epm2a^−/−^* mutant mice (Ganesh et al., 2002) was performed by targeted deletion of the fourth exon of the *Epm2a* gene (Minassian et al., 1998; Serratosa et al., 1999), which encodes the dual-specificity phosphatase domain that is critical for the function of laforin. Constitutive *Epm2b^−/−^* mutant mice were generated by targeting deletion of the unique exon encoding malin, as previously described (Criado et al., 2012). The genetic background of null-mouse lines used in the study was a mix of 129Sv:C57BL/6J (25%:75%). The mice were kept at the IIS-Fundación Jimenez Jiménez Díaz animal facility and were maintained on a 12:12-h light/dark cycle under constant temperature (23°C) with free access to regular food and water *ad libitum*. Two groups of homozygous animals aged 4–8 and 9–23 months (representative samples of young adult and aged mice) were analyzed for each experiment. The experiments were conducted in accordance with the Declaration of Helsinki principles and the guidelines of the Institutional Animal Care and Use Committee, and were approved by the IIS-Fundación Jiménez Díaz ethical review board.

### Histology and immunoshistochemistry

The animals were anesthetized with a mixture of medetomidine (Domitor, Pfizer, Espoo, Finland), ketamine (Ketolar, Pfizer, Espoo, Finland), and sterile water (1:1.5:1.5) and transcardially perfused with 4% phosphate-buffered paraformaldehyde. The animals' brains were removed, postfixed overnight at 4°C, dehydrated in graded ethanol solutions, and embedded in paraffin. They were then sectioned in serial arrays of 7-μm-thick sections, deparaffinized at 60°C, and stored at room temperature until use. For PAS staining, coronal sections were processed as previously described (Mitsuno et al., 1999), and counterstained with hematoxylin solution. For immunohistochemistry, contiguous PAS-stained sections were rehydrated and incubated in 1.5% (vol/vol) H_2_O_2_ in methanol for 20 min at room temperature to inactivate endogenous peroxidase. Sections were then placed in blocking buffer for 60 min at room temperature and incubated for 3 days at 4°C with anti-ubiquitin antibody (Dako, Glostrup, Denmark). Subsequently, the sections were treated with anti-rabbit biotinylated secondary antibody and stained using the Vectastain ABC kit (Vector Laboratories, Burlingame, CA, USA). Immunoreactivity was developed with diaminobenzidine (Dako, Glostrup, Denmark), and sections were counterstained with hematoxylin solution, dehydrated, and covered with DePeX (SERVA Electrophoresis GmbH, Heidelberg, Germany).

### PTZ treatment

PTZ (Sigma Chemicals, St. Louis, MO, USA) was dissolved in sterile water and injected intraperitoneally at different doses as a single injection. The doses administered were those known to be sub-convulsive (30 mg/kg) (Erakovic et al., 2001) and convulsive (50 mg/kg) (Erakovic et al., 2001; Eloqayli et al., 2003) for control mice. As described previously, a dose of 50 mg/kg induces seizures in 50% of control animals (Shitak et al., 2006). After drug administration, PTZ-induced myoclonic jerks and seizure monitoring was performed in 16–36 mice per group using visual blinded observation over a period of 45 min. The percentages of mice displaying myoclonic jerks and generalized tonic-clonic seizures were recorded in 3 groups of control, *Epm2a^−/−^*, and *Epm2b^−/−^* mice at 4–8 and 9–23 months of age. Following injection of PTZ, mice displayed periods of immobility and overt evidence of convulsive activity which included hyperactivity, twitching, and hyperextension of the limbs that at times progresses to generalized tonic-clonic seizures. We recorded the percentages of PTZ injected mice that present both muscular jerks, which usually started within a few minutes of PTZ injection, and generalized tonic-clonic seizures. To assess for the presence of EEG correlates, twelve mice chosen at random as representative samples of these 3 groups at 4–8 and 9–23 months underwent surgery and analysis by video-EEG after PTZ injection at convulsive doses. EEG recordings of operated mice revealed the existence of isolated spikes and spike-waves that correlate with muscular jerks. Generalized tonic-clonic seizures with low-frequency (3–6 Hz) spike-wave correlates were also observed in EEGs. Latency—or time between injection and onset of seizures—and seizure length were also analyzed for those mice treated with 50 mg/kg PTZ. In addition to measuring epileptic activity, PTZ-induced lethality was also analyzed for each drug dose.

### Video-electroencephalographic (EEG) recording

Procedures for implantation of intracranial electrodes and video-EEG recording have been previously described (Criado et al., 2012; Garcia-Cabrero et al., 2012). Briefly, custom-made stainless-steel screw electrodes were fixed to the skulls of anesthetized animals under a mixture of medetomidine, ketamine and sterile water (1:1.5:1.5). Two monopolar epidural electrodes were implanted symmetrically over the frontal cortex in front of bregma, whereas 2 ground and reference electrodes were positioned posterior to lambda. After a 4-day recovery period, synchronized video-EEG activity was recorded using computer-based systems (Easy EEG 2.1, Cadwell, CA, USA and Natus Neurowork EEG, Natus Medical Inc., San Carlos, CA, USA) while the mice moved freely.

### Statistical analysis

Values are given as means ± standard error of means (SEM) or percentages. Differences between groups were analyzed by two-tailed Student's *t*-test or Chi-square (Graph-Pad Prism 2.0). Statistical significance was considered to be reached at ^*^p < 0.05; ^**^p < 0.01; ^***^p < 0.001.

## Results

### Presence of lafora bodies in *Epm2a^−/−^* and *Epm2b^−/−^* mice

**Figure 1:**
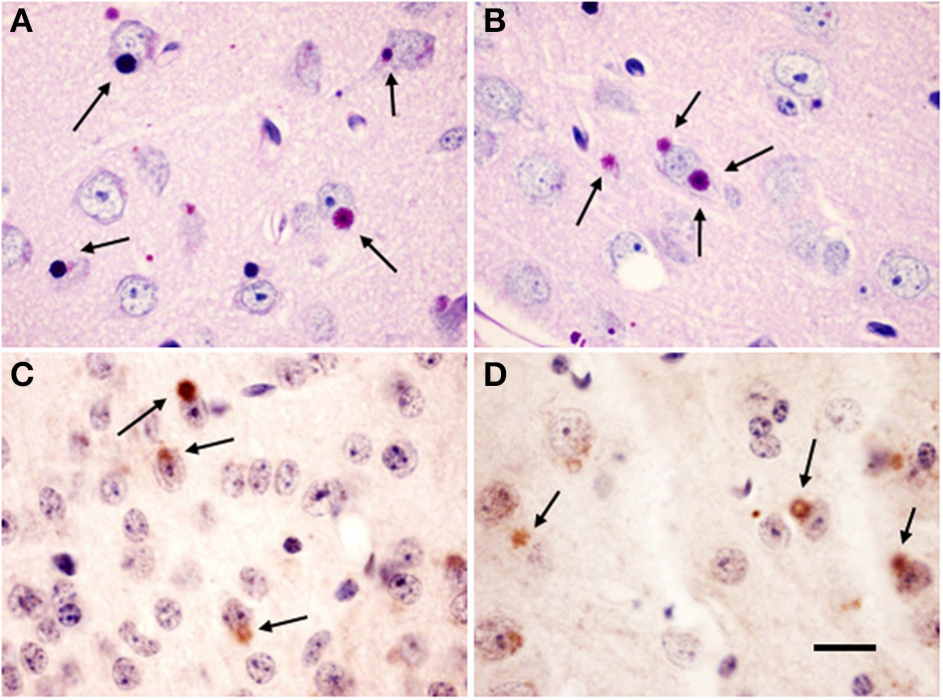
**Lafora bodies in *Epm2a^−/−^* and *Epm2b^−/−^* mice brains**. Citoplasmic polyglucosan inclusions (arrows) positive for periodic acid-Shiff staining (PAS) **(A,B)** in layer V of the cerebral cortex, and immunostained for ubiquitin **(C,D)** in Layer IV **(C)** and IV-V **(D)** of cerebral cortex of *Epm2a^−/−^* **(A,C)** and *Epm2b^−/−^* **(B,D)** mice at 17 months of age. Scale bar = 20 μm.

As in previous descriptions, Lafora inclusions were abundant in several brain regions of the *Epm2a^−/−^* and *Epm2b^−/−^* mice, including cerebral cortex, hippocampus, basal ganglia, thalamus, cerebellum, and brainstem (Criado et al., 2012; Garcia-Cabrero et al., 2012). Representative examples of neurons of Layers IV-V of the cortex exhibiting PAS-positive Lafora aggregates in cell bodies of *Epm2a^−/−^* and *Epm2b^−/−^* at 17 months of age are shown in Figures 1A,B, respectively. Intracellular inclusions immunostained for ubiquitin were also found in the same regions as PAS-positive inclusions (Figures 1C,D).

### Analysis of PTZ-induced myoclonus, generalized tonic-clonic seizures, and lethality in mice lacking laforin or malin

**Figure 2:**
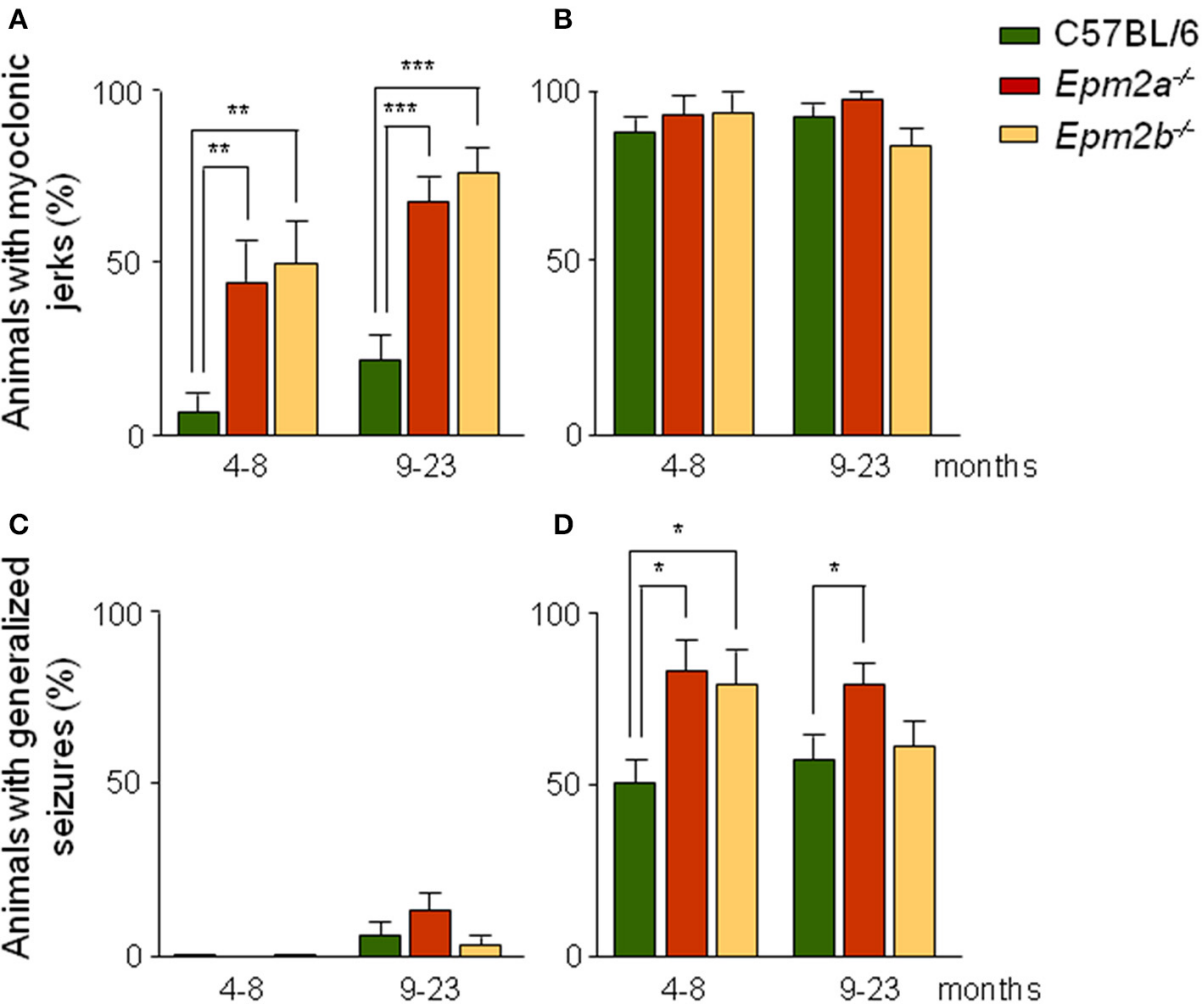
**Increased sensitivity of *Epm2a^−/−^* and *Epm2b^−/−^* models to the chemoconvulsant agent PTZ**. Two different age groups (4–8 and 9–23 months) of control, *Epm2a^−/−^*, and *Epm2b^−/−^* mice were injected intraperitoneally with 30 **(A,C)** and 50 mg/kg of PTZ **(B,D)** and the percentages of mice showing PTZ-induced myoclonus **(A,B)**, and generalized seizures **(C,D)** were recorded ^*^p < 0.05; ^**^p < 0.01; ^***^p < 0.001, Chi-square (*n* = 16–36 mice per group at each PTZ dose).

Following injection of PTZ, control mice displayed periods of immobility and overt evidence of convulsive activity which included hyperactivity, twitching, and hyperextension of the limbs, later progressing to generalized tonic-clonic seizures. We established scores for the presence of both myoclonic jerks which usually started within a few minutes of PTZ injection, and tonic-clonic seizures. EEG recordings of operated mice revealed the existence of isolated spikes and spike-wave correlates. At subconvulsive doses, the percentage of mutant mice showing myoclonic jerks was higher than that of control mice (Figure 2A). Thus, at this dose, 45% of *Epm2a^−/−^* and 50% of *Epm2b^−/−^* mice at 4–8 months displayed myoclonic jerks, compared to only 7% of age-matched control mice. In the 9-to-23-month mice group, myoclonic jerks were present in 67% of *Epm2a^−/−^* and 75% of *Epm2b^−/−^*, compared to only 22% of control mice. When the induction of myoclonic jerks was analyzed at a higher dose of PTZ (50 mg/kg), no major differences were observed between controls and *Epm2a^−/−^* or *Epm2b^−/−^* mice (Figure 2B). These results indicate that a sub-convulsive dose of the epileptogenic drug PTZ induces the appearance of myoclonic jerks more easily in both young and old mice lacking the expression of either laforin or malin proteins, as compared to age-matched controls. Although both *Epm2a^−/−^* and *Epm2b^−/−^* mice trend to present more myoclonic jerks with age, no statistical differences were found between young and older mice. After administration of subconvulsive doses of PTZ in control, *Epm2a^−/−^*, or *Epm2b^−/−^* mice at 4–8 months of age, no generalized tonic-clonic seizures were observed (Figure 2C). However, in the oldest group, a small proportion of mice displayed seizures, though no significant differences were recorded between control and mutant mice (6% control, 13% *Epm2a^−/−^*, and 3% *Epm2b^−/−^*) (Figure 2C). As expected, a convulsive PTZ dose at 4–8 months of age induced tonic-clonic seizures in 50% of control mice (Figure 2D). Interestingly, this percentage was notably increased for both *Epm2a^−/−^* (80%) and *Epm2b^−/−^* (78%) mice (Figure 2D). Similar results were obtained for *Epm2a^−/−^* (78%) mice at 9–23 months, whereas no major differences between control and *Epm2b^−/−^* mice were noticed at this age (Figure 2D). Lethality thresholds were not affected in either *Epm2a^−/−^* or *Epm2b^−/−^* mice at any PTZ dose as compared to age-matched controls (data not shown).

### PTZ-induced seizure latency and length

**Figure 3:**
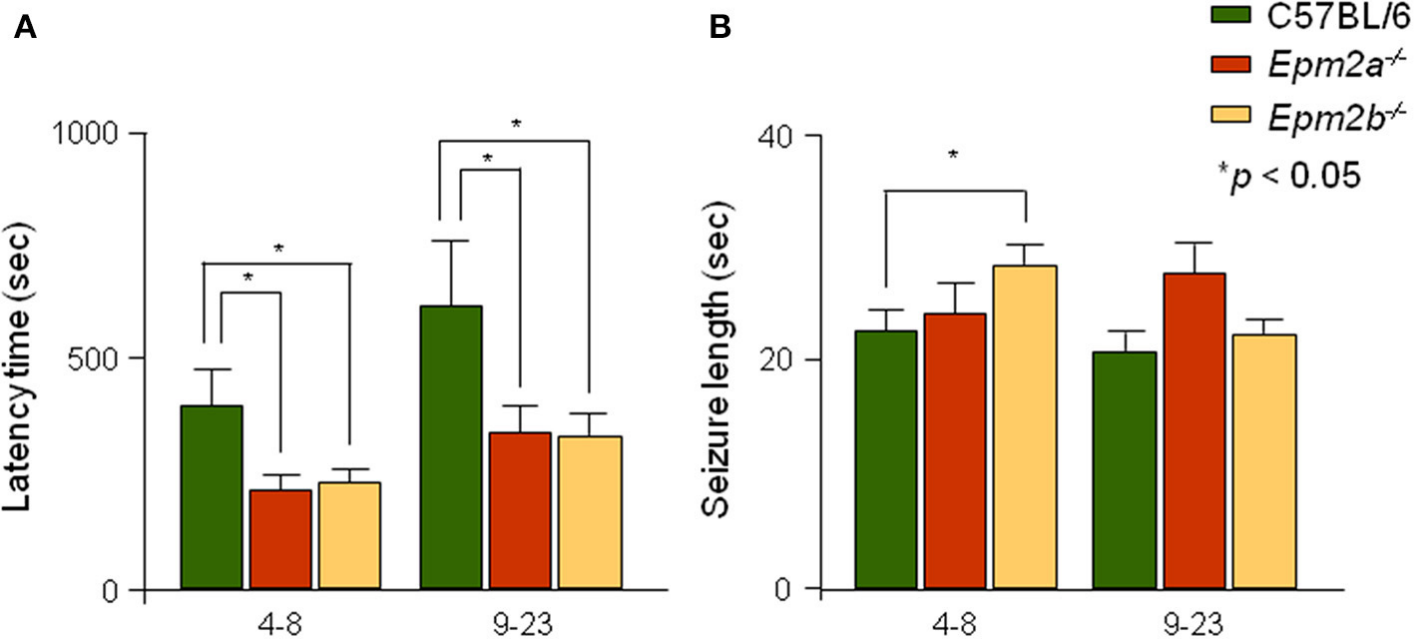
**Analysis of seizure latency and length of PTZ-induced generalized seizures in *Epm2a^−/−^* and *Epm2b^−/−^* mice**. Mice within 2 different age spans (4–8 and 9–23 months) were injected with a convulsive dose of PTZ (50 mg/kg). **(A)** The time interval between drug administration and development of generalized tonic-clonic seizures and **(B)** the seizure length were measured. Data are presented as mean ± s.e.m. Student's *t*-test was performed for statistical evaluation. ^*^p < 0.05 (*n* = 12–25).

Latency, or the time elapsed between injection of a convulsive dose of PTZ (50 mg/kg) and tonic-clonic seizure appearance, was recorded for controls, *Epm2a^−/−^*, and *Epm2b^−/−^* mice. Mutant mice in both age ranges showed statistically significant reductions in seizure latency when compared to age-matched control mice (Figure 3A). Additionally, the length of PTZ-induced seizures in the *Epm2b^−/−^* model at 4–8 months of age was significantly increased (Figure 3B). The length of PTZ-induced seizures in the *Epm2a^−/−^* mice at 9–23 months of age was also increased, although no significant differences were observed between old *Epm2a^−/−^* or *Epm2b^−/−^* mice and age-matched controls.

### Video-EEG recordings of PTZ-induced seizures in control, *Epm2a^−/−^*, and *Epm2b^−/−^* mice at 16 months of age

**Figure 4:**
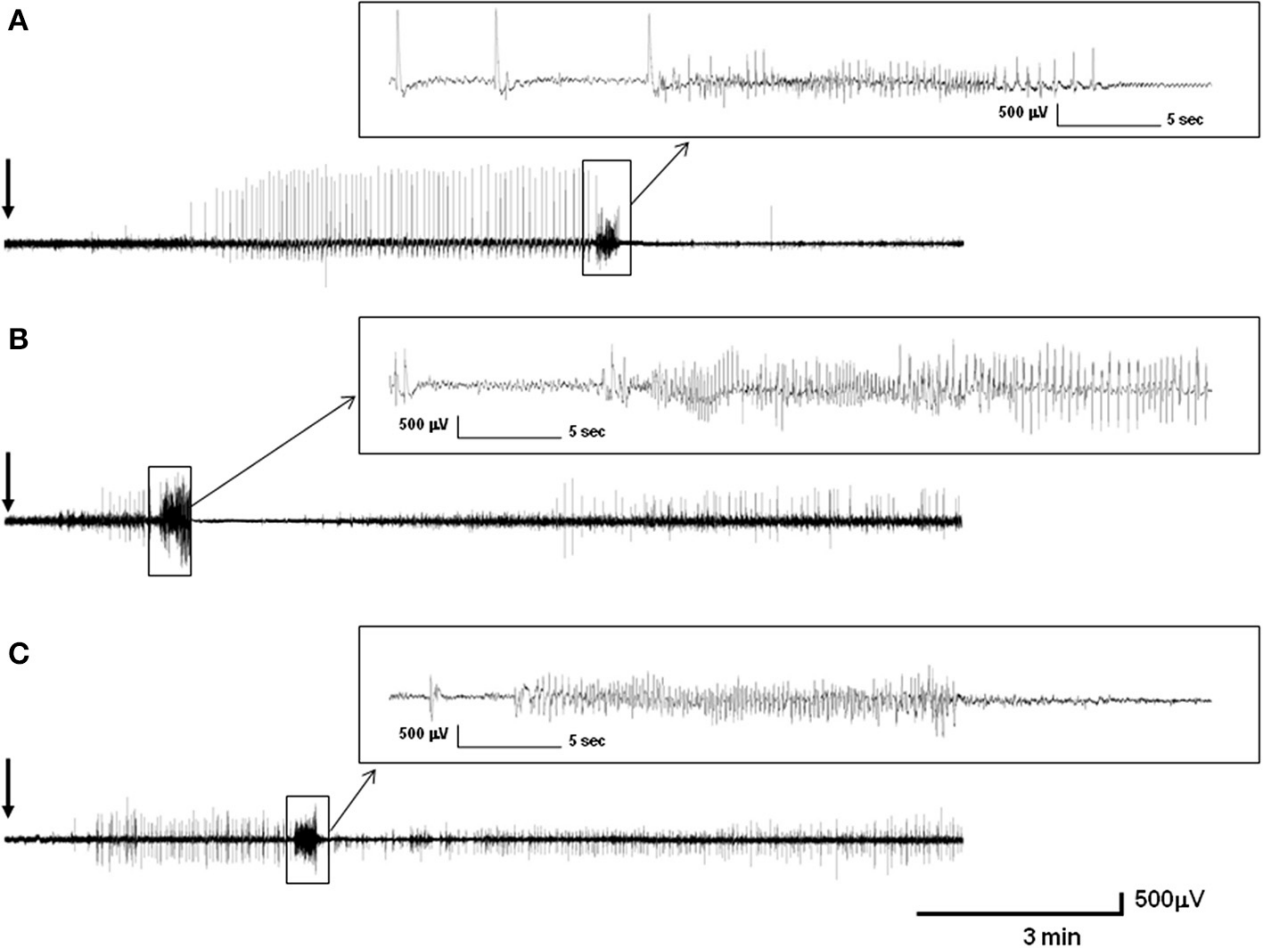
**Intracranial representative recording of control, *Epm2a^−/−^*, and *Epm2b^−/−^* mice at 16 months of age after injection of 50 mg/kg pentylenetetrazol (PTZ)**. **(A)** Electroencephalographic activity of a control mouse **(A)**, of an *Epm2a^−/−^* mouse **(B)**, and of an *Epm2b^−/−^* mouse **(C)**. The figure shows the records from monopolar electrodes placed over the left frontal cortex with the reference electrodes implanted posterior to lambda.

Injections of PTZ at convulsive doses in control mice produced periods of immobility followed by twitching and hyperextension of the limbs, further progressing to generalized tonic-clonic seizures. Intracranial EEG recordings of control, *Epm2a^−/−^*, and *Epm2b^−/−^* mice showed different specific patterns of discharges after 50-mg/kg PTZ injections. Thus, operated control mice (representative example is shown in Figure 4A) showed a pattern of discharges starting 1 min and 19 s after PTZ injection, with isolated spike and spike-wave discharges alternating with immobility periods during 3 min. At this point, rhythmic isolated spike and spike-wave discharges with muscular jerk correlates were shown over a period of 5 min; these evolved to generalized tonic-clonic seizures with low-frequency (3–6 Hz) spike-wave correlates lasting 22 s. The tonic-clonic seizure began with a tonic phase that was followed by a clonic stage and an after-seizure period of immobility. A representative EEG recording of an *Epm2a^−/−^* mouse after PTZ injection (Figure 4B) displayed a significantly shorter period of time (1 min and 30 s) before a brief 1-min initial stage of irregular twitches and myoclonus that correlated with spike and spike-wave complexes and that evolved into a tonic-clonic seizure lasting 29 s. This seizure started with a tonic phase lasting 14 s with hyperextension of the limbs and was followed by a 4-s period of clonic stage and a 24-s period of tonic hind-limb extension. The first 11 s of this tonic phase presented with spike-wave complex correlates and concluded with a 13-s period of non-neuronal activity. A representative example of EEG recording of an *Epm2b^−/−^* mice (Figure 4C) showed a 1-min period of normal activity after PTZ injection, which was followed by myoclonus, muscular jerks, and twitches with EEG correlates of spikes and spike-wave and polyspike-wave complexes over a period of 3 min, preceding a generalized tonic-clonic seizure lasting 22 s. This seizure started with a 3-s tonic phase with hyperextension of the limbs that was followed by a prominent tonic-clonic stage lasting 19 s. The post-seizure period of non-neuronal activity was almost absent in the *Epm2b^−/−^* model while post-ictal discharges with myoclonic correlates started suddenly in this model.

## Discussion

Although a great effort has been devoted to understanding the basis of the formation of Lafora bodies and their relationship to autophagy and alterations in glycogen metabolism (Gentry et al., 2007; Tagliabracci et al., 2008; Aguado et al., 2010; Criado et al., 2012), the relationships between the development of Lafora bodies and the induction of abnormal electroencephalographic activity remain unknown. Lafora disease patients present a distinctive EEG pattern characterized by slowing of background activity with recurrent epileptiform discharges: 3–6 Hz spikes/polyspikes, with or without slow waves (Van Heycop Ten Ham, 1974), similar to that observed in spontaneous seizures developed by *Epm2a^−/−^* and *Epm2b^−/−^* mice (Ganesh et al., 2002; Garcia-Cabrero et al., 2012).

It has been previously reported that the lack of expression of malin protein in a different *Epm2b^−/−^* mice model (Valles-Ortega et al., 2011) resulted in an increase in the susceptibility to pharmacologically induced hippocampal seizures using the epileptogenic kainic acid. PTZ is a widely used chemical for the induction of generalized epilepsy. It has also been extensively used to test antiepileptic drugs on laboratory animals (Stone, 1970). Although animal models based on PTZ are widely used, the mechanism by which PTZ elicits its action is not very well understood. At the molecular level, a generally accepted mechanism of PTZ is non-competitive antagonism of the gamma-aminobutyric acid GABA_A_ receptor complex. Additionally, alterations in voltage-dependent calcium and potassium channels and changes in the serotonergic and in the NMDA receptor-mediated glutamate neurotransmission are also generated after PTZ treatment. Treatment with PTZ in mice induces a wide range of responses, from mild convulsions to generalized seizures, all of them as a result of an excess of activation of neurons located in the frontal cortex, the amygdala, the cerebellum, the brainstem, and other regions of the brain.

In this study we have undertaken a systematic evaluation of PTZ-induced seizure responses in *Epm2a^−/−^* and *Epm2b^−/−^* mice. Both models present a significantly increased sensitivity to PTZ that is more noticeable in the presence of myoclonus at sub-convulsive doses. Although both *Epm2a^−/−^* and *Epm2b^−/−^* mice trend to present more myoclonic jerks with age, no statistical differences were found between young and older mice. At convulsive doses, the percentage of *Epm2a^−/−^* and *Epm2b^−/−^* mice showing generalized seizures was significantly increased, as was seizure length, while the latency time to the appearance of these seizures experienced a statistically significant reduction when compared to age-matched controls. Although old *Epm2b^−/−^* mice trended to be more resistance to PTZ than young animals, no significant differences were found between all aged groups. It has been widely reported that number of Lafora bodies increases with age in brain of *Epm2a^−/−^* and *Epm2b^−/−^* mice (Ganesh et al., 2002; Criado et al., 2012; Garcia-Cabrero et al., 2012). Moreover, alterations in motor coordination, activity impairment and memory deficits progressively increase with age in both *Epm2a^−/−^* and *Epm2b^−/−^* mice (Garcia-Cabrero et al., 2012), probably reflecting the increased accumulation of Lafora bodies in multiple brain regions. However, myoclonus and seizures were observed in both mutant mouse strains regardless of age. Thus, it seems likely that development of Lafora bodies does not interfere with the induction of abnormal electroencephalographic activity in our mouse models.

The lack of laforin and malin expression may induce some aberrant activity of neuronal networks, resulting in increased GABA_A_ receptor-mediated hyperexcitability. EEG records of PTZ-induced seizures in *Epm2a^−/−^* and *Epm2b^−/−^* mice showed the presence of characteristic patterns of discharges. Both *Epm2a^−/−^* and *Epm2b^−/−^* mice display a shorter period of latency with irregular spike and spike-wave discharges before the appearance of the tonic-clonic seizure as compared to age-matched controls. Moreover, post-ictal discharges with myoclonic correlates started suddenly in both models. This behavior may reflect the existence of alterations in the GABAergic neurotransmission which could be involved in the generation of epileptic traits.

Overall, our results show that mice lacking expression of either laforin or malin display different degrees of increased GABA_A_ receptor-mediated hyperexcitability after administration of the epiletogenic agent PTZ. Our *Epm2a^−/−^* and *Epm2b^−/−^* models may assist in understanding the way laforin and malin mutations regulate excitotoxic damage and in assaying novel agents aimed at reducing epileptic seizures in this devastating disease.

### Conflict of interest statement

The authors declare that the research was conducted in the absence of any commercial or financial relationships that could be construed as a potential conflict of interest.
